# The impact of a dog-facilitated mobile physical activity intervention on children’s social–emotional development: a randomized controlled trial

**DOI:** 10.1093/pubmed/fdaf142

**Published:** 2025-11-03

**Authors:** Michelle Ng, Emma K Adams, Kevin Murray, Carri Westgarth, Hayley Christian

**Affiliations:** The Kids Research Institute Australia, The University of Western Australia, 15 Hospital Avenue, Nedlands, Perth, WA, 6009, Australia; The Kids Research Institute Australia, The University of Western Australia, 15 Hospital Avenue, Nedlands, Perth, WA, 6009, Australia; School of Population and Global Health, The University of Western Australia, 35 Stirling Highway, Crawley, Perth, WA, 6009, Australia; School of Population and Global Health, The University of Western Australia, 35 Stirling Highway, Crawley, Perth, WA, 6009, Australia; Department of Livestock and One Health, Institute of Infection, Veterinary and Ecological Sciences, University of Liverpool, Leahurst, Chester High Road, Neston, Cheshire, CH64 7TE, UK; The Kids Research Institute Australia, The University of Western Australia, 15 Hospital Avenue, Nedlands, Perth, WA, 6009, Australia; School of Population and Global Health, The University of Western Australia, 35 Stirling Highway, Crawley, Perth, WA, 6009, Australia

**Keywords:** children, physical activity, social determinants

## Abstract

**Background:**

Dog ownership has been suggested to be positively associated with children’s physical, social, and emotional development. This study investigated the effect of a mobile health dog-facilitated physical activity intervention on young children’s social–emotional development and attachment to the family dog.

**Methods:**

150 five- to ten-year-olds with a family dog(s) participated in the PLAYCE (‘PLAY Spaces and Environments for Children’s Physical Activity’)—PAWS study, a three-armed randomized controlled trial*.* Children were randomized into either the SMS-only, SMS + pedometer, or control group for four-weeks. Parents reported children’s social–emotional development (Strengths and Difficulties Questionnaire), empathy (Young Children’s Empathy Measure), self-regulation (Fast Track Project Child Behavior Questionnaire), and attachment to the dog (Dogs and Physical Activity Tool). Linear mixed effects models examined intervention effects at one- and three-month follow-up.

**Results:**

There were no significant differences observed between intervention and control groups at one- or three-month follow-up for social–emotional development, empathy, self-regulation, or attachment to the dog (all *P*-values > 0.05).

**Conclusions:**

Larger interventions encouraging children to be physically active with their dog are required to confirm these findings and the impact of dog-facilitated physical activity interventions on child and family health and development outcomes. Longer intervention and follow-up periods are also needed.

## Introduction

Promoting regular physical activity for children’s health and wellbeing is identified as a priority by the World Health Organization (WHO).^1^ Physical activity supports healthy weight, bone health, and cardiovascular fitness, and enhances children’s cognitive, social, and emotional development.^2–4^ Establishing positive physical activity behaviours early in life is an important way to reduce the likelihood of poor physical and mental health^4^^,^^5^ and increase physical activity across the life course.^6^^,^^7^ Yet, most children do not meet the WHO recommended 60 minutes of moderate to vigorous physical activity per day.^8^

Dog ownership is common in families with children.^9–12^ An emerging body of research suggests that dog ownership can benefit children’s physical, social, and emotional development. In conjunction to the increased physical activity children accumulate from dog walking and dog play,^13^^,^^14^ children may benefit through the emotional bonds formed with their pet dog.^15^ According to relationship psychology^16^ and attachment theory,^17^ forming relationships are a fundamental component of child development. This is further highlighted in Human-Animal Interaction research which focuses on the relationships between companion animals and humans including child development.^18^ As a relatively recent and interdisciplinary field,^19^ there is potential for Human-Animal Interaction research to uncover the benefits and mechanisms through which companion animals support child development.

The single review to date on the effect of companion animals on child and adolescent development identified 22 studies between 1960 and 2016.^15^ Most studies were cross-sectional in design. The findings suggested there were positive relationships between dog ownership and children’s social–emotional development,^12^ self-esteem, autonomy, empathy, trust, self-confidence, feelings of safety, social competence,^15^^,^^20^^,^^21^ and family cohesion.^22^ The review highlighted a lack of intervention research examining the influence of the emotional bond between children and their pet dog on child development.^23^^,^^24^ Furthermore, given that both physical activity^2^ and dog ownership^12^^,^^25^ are each associated with social–emotional benefits, increasing dog-facilitated physical activity may be a plausible strategy for positively impacting children’s social–emotional development.

Few studies have investigated the impact of dog-facilitated physical activity interventions on child outcomes,^26^ and none have explored the impact of such interventions on children’s social–emotional development. Most interventions to date have examined associations between children’s social–emotional outcomes and service or therapy dogs (animal-assisted therapy) or are in specific clinical populations such as children with autism spectrum disorder^27^ and victims of violence.^28^ The only study to date to test the effect of a dog walking intervention on children’s physical activity behaviour is the Children, Parents and Pets Exercising Together (CPET) study.^29^ The CPET study (n = 28; 9–11 years old) was a 10-week pilot intervention involving home visits by a qualified animal behaviourist, phone calls and text messages to motivate and review goal progress, and information on dog walking routes and dog play activities. No significant differences were found between the intervention and control group for physical activity or weekly dog walking. This was attributed mostly to the small sample size, however families found the intervention to be acceptable and feasible.^30^ Further research is needed to understand how interactions with the family dog influence children’s social–emotional development, at a community level.

Given the prevalence of mobile devices in the community, there is considerable potential for mobile health (‘mHealth’) strategies to improve health outcomes. Such technologies provide the opportunity for interventions to be implemented widely and at low cost within the community. The PLAYCE (‘PLAY Spaces and Environments for Children’s Physical Activity’)—PAWS study tested a minimal-contact intervention sending mobile text messages (SMS) to parents to encourage their children to walk and play with their dog more. Previously we reported dog-facilitated physical activity was greater at 3-month follow up among the SMS intervention group compared to control group, though this association was not significant in fully adjusted models.^31^ We hypothesized that increased time spent physically interacting with the family dog would improve the child-dog bond, leading to better child social–emotional development. Thus, the aim of this study was to investigate the effect of the PAWS intervention on the secondary outcomes of children’s social–emotional wellbeing, empathy, self-regulation, and attachment to the family dog.

## Methods

The study was conducted between April 2019 and October 2021 in Perth, Western Australia. Full details are published in the study protocol^32^ and primary outcome (dog-facilitated physical activity) paper.^31^ The Human Research Ethics Committee of the University of Western Australia (2021/ET000105 and RA/4/1/7417) provided approval. The study is registered with the Australian New Zealand Clinical Trials Registry (ACTRN12620000288921).

### The PLAYCE—PAWS intervention

PLAYCE—PAWS was a three-armed, parallel-group, randomized controlled trial of a physical activity-based minimal intervention involving children with a family dog. Participants were randomly assigned in staggered blocks to either intervention (SMS-only or SMS + pedometer) or control groups. Parents in the intervention groups received personalized mHealth SMS message prompts three times a week to motivate and encourage them to support their child to either walk and/or play with their dog each day. The SMS + pedometer group also received a Yamax SW200 pedometer for attaching to their dog’s collar and a personalized dog steps diary for children to record the number of steps their dog did during play or walking. To facilitate dog walking and play, families were also provided information about dog friendly parks, trails, and beaches; games for children to play with their dog; and tips about how children can safely interact with their dog. The control group continued their normal routine for the duration of the study and received intervention resources at the end of the study, thereby ensuring fair access to any beneficial outcomes of the project.

### Study design and participants

Participants were recruited from an existing cohort study (PLAYCE)^33^ and the general community through various strategies such as print advertising (newspapers, school and professional association newsletters), social media (Facebook and Twitter), crowdsourcing (via institutional websites), market research and through snowball sampling.^32^ Children were eligible if they were aged between five and 10 years old with a family dog(s) which was well socialized with the child, other people and other dogs. Children with a recognized disability (physical, emotional/behavioural, or intellectual) that affected participation in physical activity were excluded. For safety reasons the dog also had to pass a dog behaviour screening questionnaire conducted with the owner over the phone by the study team.^32^ In addition, parents were required to supervise interactions between their child and dog at all times to ensure safe dog play and walking practices. This was highlighted in the study information as well as in resources provided to parents. All parents provided written informed consent for them and their child’s participation in the study. Parents completed three online surveys at baseline, 1 month post intervention and 3 months post intervention. All participating families were sent the 3-month survey even if they did not complete the 1-month survey. The intervention period was four weeks.

**Figure 1:**
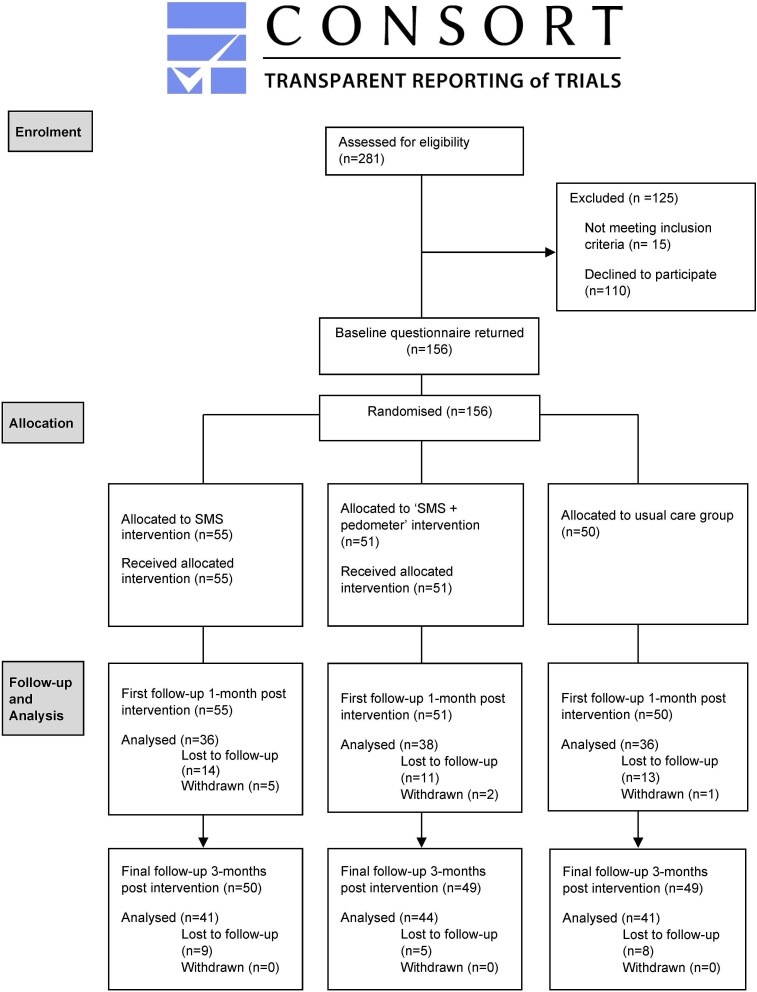
CONSORT flow diagram for randomized trial participants.

The CONSORT study flow diagram summarizes sample attrition in Fig. 1. The study complied with the CONSORT guidelines for the design and reporting a randomized trial^34^; the CONSORT checklist is provided as Supplementary Table 1.

### Sample size calculation

The sample size calculation was based on the primary outcome—child physical activity. Using PLAYCE study baseline data^33^ and CPET study data,^30^ it was assumed that a total sample size of 150 would provide 80% power to detect a one-unit difference in pre-post change in the number of times per week children did family dog walking or played with their dog between intervention and control groups. We achieved the target sample size with almost equal numbers between groups at the conclusion of the study.

### Measures

#### Children’s social–emotional development

To measure children’s social–emotional development, we used the Strengths and Difficulties Questionnaire (SDQ),^35^ the Young Children’s Empathy Measure^36^ and the Modified Child Problem Behaviour Checklist from the Fast Track Project for self-regulation.^37^ The SDQ is a validated and commonly used 25-item instrument (scale: ‘not true’, ‘somewhat true’, ‘certainly true’) that measures the social and emotional well-being of children aged 2–17 years.^35^ Items were averaged according to five subscales: emotional symptoms, conduct problems, hyperactivity-inattention, peer relationship problems, pro-social behaviour. The total difficulties score is a sum of four subscales except prosocial behaviour.^35^ The parent-report version of the SDQ has satisfactory reliability.^38^

Parents reported their child’s ability to identify sadness, fear, anger and happiness in four scenarios on the Young Children’s Empathy Measure: sadness (a child has just lost their best friend), fear (a child is chased by a big, nasty monster), anger (a child really wants to go out but is not allowed) and happiness (a child is going to its most favourite park to play).^36^ Responses were coded into an accuracy rating based on: 1 = exact match to the intended emotion or 0 = another emotion. The number of correct emotional responses were summed with a higher score indicative of higher levels of empathy. This measure has acceptable internal reliability (α = 0.69).^36^ To measure the child’s empathy towards dogs, the questions were repeated with ‘dog’ as the subject of the four statements.^36^ Empathy scores for the child and towards dogs were separately calculated.

The Modified Child Problem Behaviour Checklist from the Fast Track Project^37^ measured self-regulation. The checklist was generated using items from the Child Behaviour Checklist,^39^ the Revised Behaviour Problem Checklist^40^ and other items developed by the project’s investigators (see Lochman & The Conduct Problem Prevention Research Group^41^). The checklist includes 20 items measuring the frequency of child externalizing behaviour problems (‘none of the time, ‘some of the time’, ‘most of the time’, ‘all of the time’). The mean score was used with a lower score indicating better ability to self-regulate. This tool has demonstrated high internal consistency (α = 0.87).^41^

#### Level of attachment to dog

A seven-item measure of pet attachment from the Dogs and Physical Activity Tool (DAPA Tool) was used to measure (via parent report) the level of attachment of the child to their family dog.^42^ The tool includes a six-item pet attachment measure developed by Garrity and others which was reworded specific to dogs,^43^ as well as a single item on the relationship of the family with the dog (in this case ‘my child considers my dog to be part of the family’).^42^ Responses were based on a five-point Likert scale (strongly disagree to strongly agree). The tool has been shown to be a reliable measure of adult-reported levels of attachment to their dogs, with good to excellent ICC values (ICC = 0.65–0.92).^42^

#### Socio-demographic factors

Child (sex, age, siblings) and parent (sex, age, highest level of education, work status, and family structure) socio-demographic factors were collected using standard survey items.

### Statistical analysis

To compare differences between intervention and control groups, linear mixed effects models were used for each outcome with the baseline measure of the response variable used as a covariate, time (one-month and three-month follow-up), group (control, SMS, pedometer), and time-by-group interaction as fixed factors, and individual random effects. The outcomes were SDQ total difficulties score, individual SDQ subscale scores (hyperactivity, emotional difficulties, conduct problems, peer problems, and prosocial), empathy scores, self-regulation scores, and attachment scores. All models adjusted for baseline child age, child sex, and parental educational level. Adjusted estimated means and 95% confidence intervals for each outcome and group at the 1-month and 3-month follow-up are presented. Analyses were carried out following an intention-to-treat approach and using Stata v17.0.

## Results

### Sample characteristics

Table 1 reports baseline socio-demographic and social–emotional development outcomes. Children were on average 7.3 years old (SD 1.2), just over half were boys (57%), and the majority had siblings (75%). Ninety percent of respondent parents were female and the mean age of parents was 40 years (SD 5.7). Most parents had a university degree (67%), were in full or part-time employment (87%), and were in a married/de facto relationship (89%). Mean baseline SDQ total scores and subscale scores were generally within the normal range. On average, children were reported by parents to have moderate self-regulation, be highly attached to their dog, and have high levels of empathy towards other children and towards dogs.

**Table 1:** Baseline characteristics overall and by group.

	Total sample (n = 148)	SMS group (n = 50)	SMS + pedometer group (n = 49)	Control group (n = 49)
	***Mean (SD)***	***Mean (SD)***	***Mean (SD)***	***Mean (SD)***
Child age^a^	7.3 (1.1)	7.4 (0.9)	7.1 (1.2)	7.3 (1.3)
Parent age	40.1 (5.7)	40.5 (5.2)	39.5 (5.4)	40.2 (6.5)
	***n (%)***	***n (%)***	***n (%)***	***n (%)***
Child sex (boys)	84 (56.8)	29 (58.0)	31 (63.3)	24 (49.0)
Parent sex (female)	133 (90.0)	49 (98.0)	40 (81.6)	44 (89.8)
Parent education				
Secondary level	19 (12.8)	6 (12.0)	9 (18.4)	4 (8.2)
Trade/diploma	34 (23.0)	15 (30.0)	9 (18.4)	10 (20.4)
University/Post-graduate	95 (64.2)	29 (58.0)	31 (63.3)	35 (71.4)
Work status				
Full-time	59 (39.9)	20 (40.0)	15 (30.6)	24 (49.0)
Part-time	69 (46.6)	24 (48.0)	27 (55.1)	18 (36.7)
Not working	3 (2.0)	2 (4.0)	0 (0.0)	1 (2.0)
Home duties	17 (11.5)	4 (8.0)	7 (14.3)	6 (12.2)
Family structure				
Partnered	132 (89.2)	43 (86.0)	43 (87.8)	46 (93.9)
Single	16 (10.8)	7 (14.0)	6 (12.2)	3 (6.1)
Siblings (yes)	110 (74.3)	39 (78.0)	36 (73.5)	35 (71.4)
Social–emotional development	***Mean (SD)***	***Mean (SD)***	***Mean (SD)***	***Mean (SD)***
SDQ				
Total difficulties^b^	10.3 (6.4)	9.9 (7.0)	9.8 (5.8)	11.3 (6.4)
Emotional difficulties^c^	2.6 (2.5)	2.7 (2.6)	2.1 (2.3)	3.0 (2.6)
Conduct problems^c^	2.2 (1.9)	2.1 (2.0)	2.1 (1.9)	2.3 (1.9)
Hyperactivity^c^	4.2 (2.6)	4.2 (2.9)	4.2 (2.5)	4.3 (2.4)
Peer problems^c^	1.4 (1.6)	1.0 (1.6)	1.4 (1.6)	1.7 (1.7)
Prosocial behaviour^d^	7.9 (1.9)	8.0 (1.7)	7.6 (2.2)	8.0 (1.8)
Empathy – towards other children^e^	3.6 (0.6)	3.6 (0.6)	3.6 (0.6)	3.6 (0.5)
Empathy – towards dogs^e^	2.8 (0.6)	3.0 (0.6)	2.8 (0.6)	2.7 (0.6)
Self-regulation^f^	2.4 (0.3)	2.4 (0.4)	2.4 (0.4)	2.4 (0.3)
Attachment to dog^g^	4.4 (0.7)	4.4 (0.7)	4.3 (0.8)	4.4 (0.5)

a Child age was reported for 146 children (SMS n = 49, SMS + pedometer n = 49, Control n = 48). Range 5.0–10.7 years.

b Measured by the Strengths and Difficulties Questionnaire (SDQ), a higher score reflects a higher level of social–emotional difficulties; possible range 0–40.

c Measured by the SDQ, a higher score reflects a higher level of social–emotional difficulties; possible range 0–10.

d Measured by the SDQ, a higher score reflects more helpful prosocial behaviours; possible range 0–10.

e Measured by the Young Children’s Empathy Measure, a higher score reflects higher levels of empathy; possible range 0–4.

f Measured by the Fast Track Project Child Behavior Questionnaire, a lower score indicates better ability to self-regulate; possible range 1–4.

g Measured by the Dogs and Physical Activity Tool (DAPA Tool), a higher score indicates greater attachment to the dog; possible range 1–5.

### PAWS intervention effects

Adjusted mean total and subscale SDQ scores were similar across groups at one-month and three-month follow-up (group *P*-values all *P* > .05, Table 2). Similarly, there were no differences between groups at either follow-up point for empathy towards other children, empathy towards dogs, or self-regulation (all *P* > .05). Attachment to the family dog was slightly greater at the three-month follow-up in both intervention groups compared to control, however differences between groups did not reach statistical significance (*P* = .137). Unadjusted results are presented in Supplementary Table 2.

**Table 2:** Adjusted follow-up effects of the intervention on child social–emotional development outcomes (reference group = control).

	SMS	SMS + pedometer	Control	
**1-month**	***Mean*** ***(95% CI)***	***Mean (95% CI)***	***Mean (95% CI)***	***p-value***
Total difficulties^a^	9.1 (7.9, 10.2)	9.3 (8.2, 10.5)	9.2 (8.0, 10.4)	0.943
Emotional difficulties^b^	2.2 (1.8, 2.7)	2.0 (1.5, 2.4)	2.1 (1.6, 2.5)	0.721
Conduct problems^b^	2.0 (1.5, 2.4)	2.2 (1.7, 2.6)	1.8 (1.3, 2.3)	0.516
Hyperactivity^b^	3.3 (2.8, 3.8)	3.8 (3.3, 4.2)	3.9 (3.4, 4.4)	0.186
Peer problems^b^	1.5 (1.1, 1.9)	1.4 (1.0, 1.7)	1.5 (1.1, 1.9)	0.801
Prosocial behaviour^c^	8.1 (7.6, 8.5)	8.0 (7.6, 8.4)	7.8 (7.3, 8.2)	0.620
Empathy – towards other children^d^	3.6 (3.5, 3.8)	3.6 (3.4, 3.7)	3.6 (3.5, 3.8)	0.835
Empathy – towards dogs^d^	2.7 (2.5, 2.9)	2.9 (2.7, 3.1)	2.9 (2.7, 3.1)	0.286
Self-regulation^e^	2.4 (2.3, 2.4)	2.4 (2.3, 2.5)	2.3 (2.3, 2.4)	0.649
Attachment to dog^f^	4.7 (4.5, 4.8)	4.6 (4.4, 4.7)	4.6 (4.4, 4.7)	0.549
**3-month**	***Mean (95% CI)***	***Mean (95% CI)***	***Mean (95% CI)***	***p-value***
Total difficulties^a^	9.7 (8.5, 10.8)	8.9 (7.7, 10.0)	9.1 (7.9, 10.3)	0.614
Emotional difficulties^b^	2.0 (1.6, 2.4)	2.1 (1.6, 2.5)	2.1 (1.7, 2.6)	0.908
Conduct problems^b^	2.3 (1.9, 2.8)	2.1 (1.7, 2.5)	1.8 (1.3, 2.2)	0.260
Hyperactivity^b^	3.8 (3.4, 4.3)	3.4 (2.9, 3.9)	3.8 (3.3, 4.3)	0.373
Peer problems^b^	1.5 (1.1, 1.8)	1.2 (0.9, 1.6)	1.4 (1.0, 1.8)	0.699
Prosocial behaviour^c^	8.0 (7.6, 8.5)	8.1 (7.7, 8.5)	7.9 (7.5, 8.3)	0.820
Empathy – towards other children^d^	3.5 (3.4, 3.7)	3.7 (3.5, 3.8)	3.5 (3.3, 3.6)	0.257
Empathy – towards dogs^d^	2.8 (2.6, 2.9)	2.9 (2.7, 3.1)	3.0 (2.8, 3.2)	0.169
Self-regulation^e^	2.3 (2.3, 2.4)	2.3 (2.2, 2.4)	2.4 (2.3, 2.4)	0.779
Attachment to dog^f^	4.7 (4.5, 4.8)	4.6 (4.5, 4.7)	4.4 (4.3, 4.6)	0.137

Estimated means from LMM adjusted for child sex, child age, parental education level, and baseline value for outcome variables. P-value for between-group differences at each follow-up time point, significant p-values (<0.05) are bolded. All SDQ models n = 131, empathy models n = 130, self-regulation model n = 130, attachment to dog model n = 127.

a Measured by the Strengths and Difficulties Questionnaire (SDQ), a higher score reflects a higher level of social–emotional difficulties; possible range 0–40.

b Measured by the SDQ, a higher score reflects a higher level of social–emotional difficulties; possible range 0–10.

c Measured by the SDQ, a higher score reflects more helpful prosocial behaviours; possible range 0–10.

d Measured by the Young Children’s Empathy Measure, a higher score reflects higher levels of empathy; possible range 0–4.

e Measured by the Fast Track Project Child Behavior Questionnaire, a lower score indicates better ability to self-regulate; possible range 0–4.

f Measured by the Dogs and Physical Activity Tool (DAPA Tool), a higher score indicates greater attachment to the dog; possible range 1–5.

## Discussion

### Main finding of this study

This study investigated the effects of an mHealth dog-facilitated physical activity intervention on children’s social–emotional development. No statistically significant between group differences were observed at one- or three-month follow-up in SDQ scores, empathy, self-regulation, or attachment to the family dog. Based on the study’s theoretical model,^32^ we hypothesized that increasing family dog walking and active play would increase children’s physical activity as well as time spent interacting with the dog, both of which are beneficial for supporting child-dog attachment and child development.^32^ However, we found no evidence that a four-week mHealth dog-facilitated physical activity intervention improved children’s dog attachment or social–emotional development. It may be that longer intervention and follow-up periods are necessary, particularly when interventions are light touch mHealth interventions designed for delivery at scale.

### What is already known

There is limited research on the influence of dog-facilitated physical activity interventions on children’s health and development. Our previous research found the PAWS intervention may be effective in increasing children’s dog-facilitated physical activity.^31^ It has been suggested that increasing direct contact with pet dogs develops a stronger bond between a child and their dog.^44^ This may also support the development of various positive emotional behaviours such as empathy and prosocial behaviours in children.^44^

### What this study adds

Despite our null findings, our exploratory research adds to the growing body of literature on the effects of dog-facilitated physically activity on child social–emotional development. Some other studies have also found limited evidence of cross-sectional or longitudinal associations between dog ownership and child development,^45^^,^^46^ even when accounting for pet interactions,^45^ though whether interventions can affect these relationships is underexplored. Further research is warranted to understand the longer-term effects of increased dog-facilitated physical activity-based interactions on child social and emotional development, as well as other development outcomes such as physical and cognitive development. In addition, as our sample size calculation was based upon the expected physical activity changes rather than social-development changes, the potential effect sizes to be expected from such an intervention and how meaningful they are in the real world, require further investigation. As the strength of the human-dog bond is likely an important factor in improving health and development outcomes in dog owners,^47^ such research should investigate the mediating or moderating effects of attachment to the dog on different domains of child development. Furthermore, the impact of changes in dog ownership status over time on child physical activity and developmental outcomes requires investigation. Future research may also wish to consider the role of dog-facilitated physical activity interventions on children’s recreational sedentary screen time use. Finally, the value of family dog-facilitated interventions for parents and caregivers’ health and wellbeing should also be examined.

### Strengths and limitations

A limitation of this study was the four-week PAWS intervention was likely not sufficiently long enough to affect significant changes in children’s social–emotional development. Moreover, the measures used may not have been sensitive enough to capture changes in child social–emotional development within the three-month follow-up period and the sample size may not have been sufficient to detect significant changes in these secondary outcomes. In addition, given the intervention was targeted at parents via SMS prompting, children may not have received the direct impact of the intervention. This may have curtailed the promotion of more interaction between the child and their dog, and thus children may not have fully received the anticipated benefits of the intervention. Other strategies may be required to encourage increased interaction between children and the family dog which could enhance the child-dog bond and potentially support child development. Similarly, children’s extra-curricular activities including sport participation may have reduced the time they had available to interact physically with their dog and benefit socially-emotionally. Study strengths include the relatively large sample size for an intervention study in this field, high study retention rate (>80%), detailed and context-specific outcome measures, adjustment for demographic confounders, and two post-intervention follow-up timepoints.

## Conclusion

With a high proportion of dog owning children in the community, there is an opportunity to positively impact children’s social–emotional well-being through increased interactions with their pet dog. However, there were no evidence of significant effects of this dog-facilitated physical activity intervention on child-dog attachment nor social–emotional development. Innovative interventions focused on encouraging children to be physically active with their dog are required to understand the impacts of dog-facilitated physical activity on children’s development.

## Supplementary Material

Supplementary_Material_fdaf142

## Data Availability

The de-identified data generated from this study will be available for analytic purposes by academics, research staff and students if approved by the Principal Investigator and relevant ethics committees, from 12 months following the anticipated project end date.

## Notes

**Michelle Ng,** MPH, Ms

**Emma K. Adams,** MPH, Ms

**Kevin Murray,** PhD, Associate Professor

**Carri Westgarth,** PhD, Professor

**Hayley Christian,** PhD, Professor
