# Supplementary material for: The impact of a dog-facilitated mobile physical activity intervention on children’s social–emotional development: a randomized controlled trial
Source: J Public Health (Oxf). 2025 Nov 3;48(1):144–53. doi: 10.1093/pubmed/fdaf142 (PMC13017615; doi:10.1093/pubmed/fdaf142)
Supplement: Supplementary_Material_fdaf142 [file supplementary_material_fdaf142.docx]

# Supplementary Material

Table I: CONSORT checklist

| **Section/Topic** | **Item No** | **Checklist item** | **Reported on page No** |
| --- | --- | --- | --- |
| **Title and abstract** | | | |
|  | 1a | Identification as a randomised trial in the title | 1 |
|  | 1b | Structured summary of trial design, methods, results, and conclusions (for specific guidance see CONSORT for abstracts) | 2 |
| **Introduction** | | | |
| Background and objectives | 2a | Scientific background and explanation of rationale | 3-4 |
|  | 2b | Specific objectives or hypotheses | 4 |
| **Methods** | | | |
| Trial design | 3a | Description of trial design (such as parallel, factorial) including allocation ratio | 5 |
|  | 3b | Important changes to methods after trial commencement (such as eligibility criteria), with reasons | n/a |
| Participants | 4a | Eligibility criteria for participants | 5 |
|  | 4b | Settings and locations where the data were collected | 4-5 |
| Interventions | 5 | The interventions for each group with sufficient details to allow replication, including how and when they were actually administered | 5 |
| Outcomes | 6a | Completely defined pre-specified primary and secondary outcome measures, including how and when they were assessed | 6-7 |
|  | 6b | Any changes to trial outcomes after the trial commenced, with reasons | n/a |
| Sample size | 7a | How sample size was determined | 6 |
|  | 7b | When applicable, explanation of any interim analyses and stopping guidelines | n/a |
| Randomisation: |  |  |  |
| Sequence generation | 8a | Method used to generate the random allocation sequence | 5-7 |
|  | 8b | Type of randomisation; details of any restriction (such as blocking and block size) | 5-7 |
| Allocation concealment mechanism | 9 | Mechanism used to implement the random allocation sequence (such as sequentially numbered containers), describing any steps taken to conceal the sequence until interventions were assigned | 5-7 |
| Implementation | 10 | Who generated the random allocation sequence, who enrolled participants, and who assigned participants to interventions | 5-7 |
| Blinding | 11a | If done, who was blinded after assignment to interventions (for example, participants, care providers, those assessing outcomes) and how | n/a |
|  | 11b | If relevant, description of the similarity of interventions | n/a |
| Statistical methods | 12a | Statistical methods used to compare groups for primary and secondary outcomes | 7 |
|  | 12b | Methods for additional analyses, such as subgroup analyses and adjusted analyses | n/a |
| **Results** | | | |
| Participant flow (a diagram is strongly recommended) | 13a | For each group, the numbers of participants who were randomly assigned, received intended treatment, and were analysed for the primary outcome | Figure I |
|  | 13b | For each group, losses and exclusions after randomisation, together with reasons | Figure I |
| Recruitment | 14a | Dates defining the periods of recruitment and follow-up | 4 |
|  | 14b | Why the trial ended or was stopped | n/a |
| Baseline data | 15 | A table showing baseline demographic and clinical characteristics for each group | Table I |
| Numbers analysed | 16 | For each group, number of participants (denominator) included in each analysis and whether the analysis was by original assigned groups | Figure I, Table II |
| Outcomes and estimation | 17a | For each primary and secondary outcome, results for each group, and the estimated effect size and its precision (such as 95% confidence interval) | Table II |
|  | 17b | For binary outcomes, presentation of both absolute and relative effect sizes is recommended | n/a |
| Ancillary analyses | 18 | Results of any other analyses performed, including subgroup analyses and adjusted analyses, distinguishing pre-specified from exploratory | n/a |
| Harms | 19 | All important harms or unintended effects in each group (for specific guidance see CONSORT for harms) | n/a |
| **Discussion** | | | |
| Limitations | 20 | Trial limitations, addressing sources of potential bias, imprecision, and, if relevant, multiplicity of analyses | 9-10 |
| Generalisability | 21 | Generalisability (external validity, applicability) of the trial findings | 8-10 |
| Interpretation | 22 | Interpretation consistent with results, balancing benefits and harms, and considering other relevant evidence | 8-10 |
| **Other information** | | |  |
| Registration | 23 | Registration number and name of trial registry | 5 |
| Protocol | 24 | Where the full trial protocol can be accessed, if available | 4 |
| Funding | 25 | Sources of funding and other support (such as supply of drugs), role of funders | 11 |

Table II: Unadjusted child social-emotional development outcomes by group at baseline, 1-month, and 3-month follow-ups

|  | **Baseline** | | | **1-month** | | | **3-month** | | |
| --- | --- | --- | --- | --- | --- | --- | --- | --- | --- |
|  | **SMS group** | **SMS + pedometer group** | **Control group** | **SMS group** | **SMS + pedometer group** | **Control group** | **SMS group** | **SMS + pedometer group** | **Control group** |
|  | ***Mean (SD)*** | ***Mean (SD)*** | ***Mean (SD)*** | ***Mean (SD)*** | ***Mean (SD)*** | ***Mean (SD)*** | ***Mean (SD)*** | ***Mean (SD)*** | ***Mean (SD)*** |
| SDQ |  |  |  |  |  |  |  |  |  |
| Total difficulties^a^ | 9.9 (7.0) | 9.8 (5.8) | 11.3 (6.4) | 8.7 (6.3) | 8.6 (5.4) | 9.8 (6.3) | 8.9 (6.5) | 8.6 (6.4) | 10.3 (6.1) |
| Emotional difficulties^b^ | 2.7 (2.6) | 2.1 (2.3) | 3.0 (2.6) | 2.4 (2.6) | 1.6 (1.7) | 2.2 (2.3) | 2.0 (2.4) | 1.9 (2.2) | 2.5 (2.4) |
| Conduct problems^b^ | 2.1 (2.0) | 2.1 (1.9) | 2.3 (1.9) | 1.8 (1.7) | 2.1 (2.2) | 1.9 (1.8) | 2.3 (2.0) | 2.0 (2.5) | 2.1 (1.8) |
| Hyperactivity^b^ | 4.2 (2.9) | 4.2 (2.5) | 4.3 (2.4) | 3.2 (2.5) | 3.7 (2.6) | 4.0 (2.7) | 3.5 (2.6) | 3.5 (2.5) | 4.0 (2.5) |
| Peer problems^b^ | 1.0 (1.6) | 1.4 (1.6) | 1.7 (1.7) | 1.3 (1.9) | 1.2 (1.5) | 1.7 (1.6) | 1.2 (1.4) | 1.3 (1.5) | 1.7 (1.6) |
| Prosocial behaviour^c^ | 8.0 (1.7) | 7.6 (2.2) | 8.0 (1.8) | 8.4 (1.5) | 7.8 (2.0) | 7.8 (1.9) | 8.2 (1.6) | 7.9 (2.0) | 7.9 (1.8) |
| Empathy – towards other children^d^ | 3.6 (0.6) | 3.6 (0.6) | 3.6 (0.5) | 3.6 (0.5) | 3.6 (0.6) | 3.6 (0.5) | 3.6 (0.6) | 3.6 (0.5) | 3.5 (0.6) |
| Empathy – towards dogs^d^ | 3.0 (0.6) | 2.8 (0.6) | 2.7 (0.6) | 2.7 (0.6) | 2.9 (0.7) | 2.8 (0.6) | 2.8 (0.6) | 2.9 (0.7) | 3.0 (0.6) |
| Self-regulation^e^ | 2.4 (0.4) | 2.4 (0.4) | 2.4 (0.3) | 2.4 (0.4) | 2.4 (0.3) | 2.4 (0.3) | 2.3 (0.4) | 2.3 (0.3) | 2.4 (0.3) |
| Attachment to dog^f^ | 4.4 (0.7) | 4.3 (0.8) | 4.4 (0.5) | 4.7 (0.4) | 4.6 (0.4) | 4.6 (0.5) | 4.7 (0.4) | 4.6 (0.4) | 4.5 (0.7) |

^a^ Measured by the Strengths and Difficulties Questionnaire (SDQ), a higher score reflects a higher level of social-emotional difficulties; possible range 0-40.

^b^ Measured by the SDQ, a higher score reflects a higher level of social-emotional difficulties; possible range 0-10.

^c^ Measured by the SDQ, a higher score reflects more helpful prosocial behaviours; possible range 0-10.

^d^ Measured by the Young Children’s Empathy Measure, a higher score reflects higher levels of empathy; possible range 0-4.

^e^ Measured by the Fast Track Project Child Behavior Questionnaire, a lower score indicates better ability to self-regulate; possible range 0-4.

^f^ Measured by the Dogs and Physical Activity Tool (DAPA Tool), a higher score indicates greater attachment to the dog; possible range 1-5.
